# *Listeria monocytogenes* bacteremia mimicking the systemic metastasis of adrenal cancer: a case report

**DOI:** 10.1186/s12879-022-07771-y

**Published:** 2022-10-15

**Authors:** Yuki Hatakeyama, Sho Nakakubo, Hirotaka Kusaka, Naomi Watanabe, Yukinori Yoshida, Hitoshi Shinzaki, Hiromitsu Hiroumi, Naoki Kishida, Satoshi Konno

**Affiliations:** 1 Department of Respiratory Medicine, Faculty of Medicine, Hokkaido University, North 15 West 7, Kita-ku, Sapporo, 060-8638 Japan; 2 Department of Internal Medicine, Sunagawa City Medical Center, Sunagawa, 073-0196 Japan; 3 Department of Respiratory Medicine, Sunagawa City Medical Center, Sunagawa, 073-0196 Japan; 4 Graduate School of Medicine, Hokkaido University, Sapporo, 060-8638 Japan

**Keywords:** *Listeria monocytogenes*, Adrenal gland, Abscess, Cancer, Case report

## Abstract

**Background:**

*Listeria monocytogenes* is a causative agent of food poisoning and is also known to cause invasive diseases, such as bacteremia, meningitis, and encephalitis, in neonates, elderly and immunocompromised patients. However, the clinical course of a multi-organ disseminated disease secondary to bacteremia has been rarely reported.

**Case presentation:**

A 76-year-old woman undergoing immunosuppressive therapy for rheumatoid arthritis presented to our outpatient clinic with a chief complaint of weight loss. Computed tomography showed a left adrenal mass, enlarged lymph nodes, and multiple intrahepatic nodules. Positron emission tomography demonstrated accumulation of fluorodeoxyglucose F18 in the adrenal mass, lymph nodes, hepatic nodules, and bones, leading to the suspicion of systemic metastasis of adrenal cancer. She subsequently developed a fever. Blood culture results led to the diagnosis of *Listeria monocytogenes* bacteremia. Percutaneous needle biopsy of the adrenal lesion revealed no malignant findings. After extended treatment with antimicrobial agents, the fever resolved, along with the disappearance of the systemic lesions.

**Conclusions:**

This case shows that listeriosis can lead to lesions in the adrenal gland, which can exhibit clinical presentation that is difficult to differentiate from malignancy on imaging studies.

## Background

*Listeria monocytogenes* is a Gram-positive bacterium that is ubiquitous in the environment, including the soil and water, and usually causes gastroenteritis after ingestion of contaminated food [1]. Among elderly, immunocompromised and neonates, it can cause invasive diseases, such as bacteremia, meningitis, and encephalitis [2]. In addition, rare cases of local lesions, such as liver abscess, osteomyelitis, and perianal abscess, have been reported [3–5]. However, the clinical course of a disseminated disease secondary to bacteremia and involving multiple organs has rarely been described. Particularly, there have been no previous reports of abscess lesions in the adrenal gland. Herein, we report a case of *L. monocytogenes* bacteremia with formation of lesions in multiple organs, mainly in the adrenal gland, which was resolved with antibiotic treatment.

## Case presentation

**Fig. 1:**
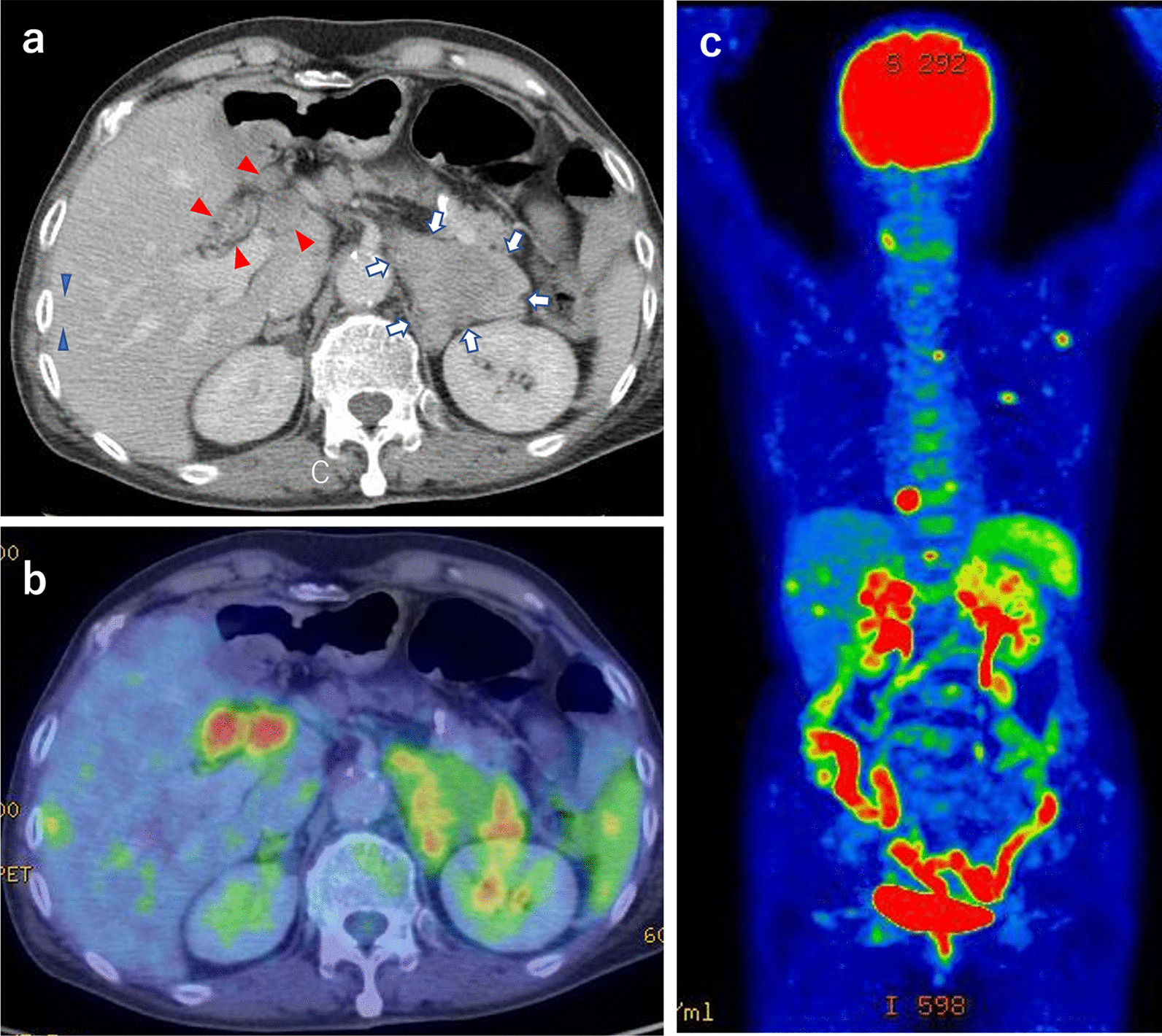
Imaging findings of the patient. **a** Contrast-enhanced computed tomography images. The image shows a mass in the adrenal gland and the enlarged lymph nodes and liver lesions. Arrows indicate the mass in the adrenal gland. Red and blue arrowheads indicate the enlarged lymph nodes and liver lesions, respectively. **b**, **c** Positron-emission tomography. Positron-emission tomography shows fluorodeoxyglucose F18 accumulation in the left adrenal gland; lymph nodes around the renal artery and common hepatic artery; and multiple nodules in the right lobe of the liver, left scapula, left sixth rib, and left lateral process of the ninth thoracic vertebra

A 76-year-old woman visited a primary care hospital with a chief complaint of 10-kg weight loss over 1 year. She had been diagnosed with rheumatoid arthritis at the age of 74 years and was being treated with methotrexate 8 mg/week, prednisolone 5 mg/day, iguratimod 25 mg/day, and golimumab 50 mg/month. Abdominal ultrasound revealed multiple nodules in the liver; thus, she was referred to our hospital. Computed tomography (CT) revealed a 60-mm mass in the left adrenal gland along with enlarged lymph nodes around the renal artery and common hepatic artery and multiple nodules in the right lobe of the liver (Fig. 1a). Positron emission tomography (PET) showed increased accumulation of fluorodeoxyglucose F18 in the same area as the lesions seen on CT, as well as in the left scapula, left sixth rib, and left lateral process of the ninth thoracic vertebra (Fig. 1b, c). Based on these findings, the radiologist suspected adrenal cancer with multiple metastases throughout the body. Thereafter, she developed a fever; thus, two sets of blood cultures were obtained. Samples were incubated with BACTEC FX (Becton Dickinson Company, USA) and three days later, Gram-positive rods were detected. Bacteria were subcultured with Trypticase Soy Agar with 5% Sheep Blood and then identified as *L. monocytogenes* using MicroScan WalkAway96plus (Beckman Coulter Inc, USA). The patient was admitted to our hospital for further investigation and treatment of the bacteremia. On admission, apart from fever, no other symptoms were noted, including diarrhea, nausea, and vomiting. She had clear consciousness. Her temperature was 39.9 °C, blood pressure was 105/67 mmHg, pulse rate was 92 beats per minute, and oxygen saturation was 97% in room air. Laboratory examination revealed anemia (hemoglobin 8.4 g/dL), thrombocytopenia (platelets 9.0 × 10^4^/µL), an elevated inflammatory response (C-reactive protein 10.6 mg/dL), hyponatremia (sodium, 134 mEq/L), and hypokalemia (potassium 2.4 mEq/L). There were no abnormal findings on other blood cell counts, biochemical tests, and spinal fluid examination. For *L. monocytogenes* bacteremia, an intravenous infusion of ampicillin (2 g every 6 h) was started on the first day of admission (day 1). On day 4, lower gastrointestinal endoscopy showed evidence of terminal ileitis, with the biopsy showing inflammatory cell infiltration with histiocytes, lymphocytes, and plasma cells. Due to the drug eruption, the antimicrobial agent was changed to sulfamethoxazole (1600 mg/day)/trimethoprim (320 mg/day) on day 19. Thereafter, gradual improvement was evident with regards fever, inflammatory response, and thrombocytopenia, which were thought to have been caused by bacteremia. However, CT imaging on day 26 demonstrated no change in the adrenal gland lesions or any other systemic lesions; hence, percutaneous needle biopsy was performed on the adrenal lesion on day 35. Histopathological examination revealed infiltration of lymphocytes and histiocytes without any malignant findings. Based on the histopathological results, we decided to continue the antimicrobial therapy, believing that the patient’s diagnosis was not adrenal carcinoma but changes associated with listeriosis. A CT scan on day 76 showed shrinkage of the adrenal and other lesions, and a CT scan on day 116 confirmed complete disappearance of the lesions. Antimicrobial therapy was discontinued on day 120, and no recurrence of fever or abscess formation has been observed. The patient has fully recovered and is continuing to visit our hospital for treatment of rheumatoid arthritis to date.

## Discussion and conclusions

Herein, we have described the rare clinical course of listeriosis. Two important insights were gained from this case: first, listeriosis can cause lesion formation in the adrenal glands; second, its clinical presentation may be difficult to differentiate from that of malignancy, on imaging studies.

Invasive listeriosis occurs at a frequency of 3–6 cases per 100,000 population, and in Japan, where this case occurred, the frequency is estimated at 6.5 cases/100,000 people [6–8]. Although it occurs more frequently in the elderly and in patients with underlying diseases, most cases present with a clinical picture of meningitis or bacteremia, and cases of space-occupying lesions in specific organs are rare. Patients with rheumatoid arthritis are known to be at a high risk of developing and dying from listeriosis, with the clinical picture being mainly bacteremia and meningitis, followed by arthritis and osteomyelitis [9, 10]. To the best of our knowledge, there have been no previous reports of abscess formation in the adrenal gland, as in the present case.

*L. monocytogenes* can invade the intestinal tract and enter the bloodstream, where it is mostly trapped by the Kupffer cells of the liver and spleen [11]. Some bacteria survive, thus leading to bacteremia and meningitis, and rarely to localized lesions. In this patient, lower gastrointestinal endoscopy showed evidence of ileitis. Since the adrenal gland contained the largest lesion, we speculated that *Listeria* entered the bloodstream from the intestinal tract and formed a lesion in the adrenal gland followed by the spread to the liver and bone. In response to *Listeria* invasion, the host induces activation of immune cells, mainly macrophages, via interleukin-18 and interferon gamma (IFN-γ) [12, 13]. The immune response of the T-cells is essential for the production of IFN-γ and for the activation of macrophages, and the reduced functioning of macrophages leads to increased susceptibility to *Listeria* [14]. Biological agents and immunosuppressive drugs used in this patient likely resulted in a markedly reduced cellular immune response; this may have led to a reduction in the ability of macrophages to eliminate organ-infiltrating *Listeria*, resulting in an increase in local abscess lesions.

In our case, *Listeria* bacteremia was confirmed; with continued antimicrobial therapy, the systemic lesions gradually shrank and eventually disappeared. Although the biopsy tissue did not confirm the organism because antimicrobial agents had already been administered, the clinical course clearly indicated that the patient had a unifying condition caused by *L. monocytogenes*.

In this case, the listeriosis resulted in the formation of a mass in the adrenal gland. Usually, mass formation in the adrenal gland is asymptomatic and discovered incidentally, and the cause is usually a noninfectious disease such as adrenal adenoma, cancer metastasis, pheochromocytoma, or hematoma [15]. The infections that can involve the adrenal gland are diverse, including viral, fungal, bacterial, and parasitic infections [16]. Space-occupying lesions such as abscesses, however, are rare. Histoplasmosis, paracoccidioides, and tuberculosis are known to produce granulomatous lesions in the adrenal glands, and some other infections such as disseminated nocardia have been reported to cause adrenal lesions [16, 17]. The initial diagnosis in this case was systemic metastasis of adrenal carcinoma. The fact that adrenal carcinoma is difficult to distinguish from abscesses on imaging [15] and that there were findings in the liver and bone on PET-CT may have accounted for the patient's listeriosis being a great mimicker of malignancy. Actinomycosis, nocardiosis, and mycosis have been reported to form foci reminiscent of malignancy, but all of these diseases are chronic infections [18–21]. We found only one report of multiple liver abscesses in an asymptomatic patient [22], but most listeriosis patients have symptoms such as fever, and follow an acute course. Our patient reported weight loss, but the cause was unclear; thus, it was not known how long the listeriosis had existed. There may be patients who develop slowly progressive listeriosis with few symptoms; however, further accumulation of such cases is necessary.

In conclusion, we encountered a case of listeriosis that had to be differentiated from adrenal carcinoma with multiple metastases. The patient was successfully diagnosed with listeriosis due to the proper collection of blood cultures prior to antimicrobial administration and absence of malignant findings on biopsy. Aggressive biopsy and microbiological examination should be considered in the presence of a space-occupying lesion of undetermined cause, especially in immunocompromised patients.

## Data Availability

The datasets used during the current study are available from the corresponding author upon reasonable request.

## Abbreviations

CT: Computed tomography
IFN-γ: Interferon gamma
PET: Positron emission tomography
